# Comment on Benekos et al. Intraocular Pressure Reduction Following Phacoemulsification in Patients with Exfoliation: A Systematic Review and Meta-Analysis. *J. Clin. Med.* 2024, *13*, 6774

**DOI:** 10.3390/jcm14082567

**Published:** 2025-04-09

**Authors:** Filippo Confalonieri, Vanessa Ferraro, Gianmaria Barone, Alessandro Gaeta, Alessandra Di Maria

**Affiliations:** 1 Department of Ophthalmology, IRCCS Humanitas Research Hospital, Rozzano, 20089 Milan, Italy; vanessa.ferraro@humanitas.it (V.F.); gianmaria.barone@humanitas.it (G.B.); alessandra.di_maria@humanitas.it (A.D.M.); 2 Department of Internal Medicine and Medical Specialties (DIMI), Università di Genova, Viale Benedetto XV, 16132 Genova, Italy; alessandrogaeta01@gmail.com

We read with great interest the recently published systematic review and meta-analysis by Benekos et al., titled “Intraocular Pressure Reduction Following Phacoemulsification in Patients with Exfoliation: A Systematic Review and Meta-Analysis” [1]. This study provides valuable insights into the intraocular pressure (IOP) reduction achieved through phacoemulsification in patients with exfoliation syndrome (XFS) or exfoliative glaucoma (XFG), a topic of considerable relevance in the field of ophthalmology.

The authors report a mean IOP reduction of 3.43 mmHg at six months and 2.75 mmHg at 12 months postoperatively. These findings reinforce the potential of cataract surgery as a therapeutic intervention for managing elevated IOP in exfoliation patients. However, we would like to draw attention to certain aspects of their analysis and discuss its relevance in light of our recent findings on preoperative IOP management with mannitol infusion.

In our study, Di Maria et al. [2], we demonstrated that preoperative intravenous mannitol significantly reduces IOP in both phakic and pseudophakic eyes, irrespective of changes in anterior chamber depth (ACD) or axial length (AXL). This suggests that optimizing preoperative IOP control through pharmacological means may complement surgical strategies such as phacoemulsification, particularly in patients with pre-existing ocular comorbidities like XFS or XFG.

Although the meta-analysis by Benekos et al. underscores the efficacy of phacoemulsification in reducing IOP, the reported GRADE evaluation reveals very low certainty of evidence due to the limited number of randomized controlled trials (RCTs) and potential biases in observational studies. Our study highlights the importance of addressing such gaps by incorporating robust, prospective methodologies that evaluate both surgical and pharmacological interventions for IOP control. Furthermore, the additive effects of preoperative IOP reduction, such as those achieved with mannitol, should be explored as a means to improve surgical outcomes and minimize postoperative IOP spikes.

We also note that the included studies in the meta-analysis primarily focused on uncomplicated phacoemulsification cases. This approach, while methodologically sound, might not fully reflect the complexity of real-world clinical scenarios where patients with XFS or XFG often present with advanced disease and higher surgical risks. Incorporating preoperative measures, such as mannitol administration, could offer additional safety and efficacy benefits in such populations.

Potential risks of mannitol infusion include fluid and electrolyte imbalances, acute kidney injury, and intracranial pressure rebound, which should be carefully monitored [3].

In conclusion, while the findings by Benekos et al. provide a solid foundation for understanding the role of phacoemulsification in IOP reduction, future research should aim to integrate pharmacological and surgical approaches to optimize outcomes for exfoliation patients. We believe that combining evidence from systematic reviews with data from prospective interventional studies, like ours, can help establish comprehensive protocols for the management of IOP in this challenging patient cohort.

We commend the authors for their significant contribution to the literature and look forward to further advancements in this field.
